# Expansion of off-site percutaneous coronary intervention centres significantly reduces ambulance driving time to primary PCI in the Netherlands

**DOI:** 10.1007/s12471-020-01466-2

**Published:** 2020-07-20

**Authors:** N. P. G. Hoedemaker, R. J. de Winter, G. J. Kommer, H. Giesbers, R. Adams, S. E. van den Bosch, P. Damman

**Affiliations:** 1 Heart Centre, Department of Cardiology, Onze Lieve Vrouwe Gasthuis, Amsterdam, The Netherlands; 2 Heart Centre, Department of Cardiology, Amsterdam Cardiovascular Sciences, Amsterdam University Medical Center, AMC/University of Amsterdam, Amsterdam, The Netherlands; 3 National Institute for Public Health and the Environment (RIVM), Bilthoven, The Netherlands; 4 Department of Cardiology, Radboud University Medical Centre, Nijmegen, The Netherlands

**Keywords:** STEMI, Off-site PCI, Time delay

## Abstract

**Introduction:**

In patients with ST-elevation myocardial infarction (STEMI), percutaneous coronary intervention (PCI)-mediated reperfusion is preferred over pharmacoinvasive reperfusion with fibrinolysis if transfer to a PCI centre can be ensured in ≤120 min. We evaluated the ambulance driving time to primary PCI centres in the Netherlands and assessed to what extent ambulance driving times were impacted by the expansion of off-site PCI centres.

**Methods and results:**

We calculated the driving routes from every Dutch postal code to the nearest PCI centre with (on-site) or without (off-site) surgical back-up. We used data from ambulance records to estimate the ambulance driving time on each route. There were 16 on-site and 14 off-site PCI centres. The median (interquartile range) time to on-site PCI centres was 18.8 min (12.2–26.3) compared with 14.9 min (8.9–20.9) to any PCI centre (*p* < 0.001). In postal code areas that were impacted by the initiation of off-site PCI, the median driving time decreased from 25.4 (18.2–33.1) to 14.7 min (8.9–20.9) (*p* < 0.001). Ambulance driving times of >120 min were only seen in non-mainland areas.

**Conclusion:**

Based on a computational model, timely ambulance transfer to a PCI centre within 120 min is available to almost all STEMI patients in the Netherlands. Expansion of off-site PCI has significantly reduced the driving time to PCI centres.

## What’s new?

- European guidelines recommend transfer to a percutaneous coronary intervention (PCI) centre if primary PCI can be ensured in ≤120 min.
- The expansion of off-site PCI centres in the Netherlands has significantly reduced the driving time to PCI centres.
- Timely ambulance transfer to a PCI centre within 120 min is available to all patients with ST-elevation myocardial infarction living on the Dutch mainland.

## Introduction

Percutaneous coronary intervention (PCI) is a commonly used treatment strategy for coronary revascularisation in both stable coronary artery disease and acute coronary syndrome (ACS) [1]. In the past 20 years, PCI has become a cornerstone in ACS treatment, particularly in patients with ST-segment elevation myocardial infarction (STEMI) [2]. Timely treatment plays an important role in the prognosis of STEMI patients [3]. In STEMI patients with symptoms for <12 h, PCI-mediated reperfusion (wire crossing) is preferred over pharmacoinvasive reperfusion with fibrinolysis if transfer to a PCI centre can be ensured in ≤120 min [4]. In addition, the recommended time from STEMI diagnosis to wire crossing during PCI is ≤90 min [4]. Several measures have been taken to reduce time delays, including field triage, transmission of prehospital electrocardiography (ECG), collaboration of healthcare providers in a STEMI-network, and feedback reporting on time intervals [3, 5–7].

In its early days, PCI was only performed in hospitals with on-site back-up for emergency cardiac surgery [8]. However, during the last 10–15 years, the procedure has been expanded to include hospitals without on-site cardiac surgery (off-site PCI). Expansion of off-site PCI centres ensures timely revascularisation in STEMI patients living in remote areas, which was observed in the United States and the United Kingdom (UK) [9–12]. In the Netherlands, the first off-site primary PCI centre was established in 2003 [13]. In 2008, the Dutch government eased the regulations on permits for hospitals to perform specific cardiac interventions in order to anticipate the future need for PCI, after which the number of off-site PCI centres further increased [14]. To keep their permits, off-site PCI centres are required to perform a minimum of 600 PCI procedures annually.

Currently, there are 30 PCI centres in the Netherlands, of which 14 are off-site PCI centres. However, it remains unknown to what extent the expansion of off-site PCI centres has impacted ambulance driving times for STEMI patients on a national scale. We evaluated the ambulance driving time to a centre for primary PCI care in the Netherlands. In addition, we investigated to what degree off-site PCI centres have contributed to a reduction in ambulance driving times.

## Methods

### Study design

We conducted a study assessing the accessibility of primary PCI care in the Netherlands, measured by ambulance driving time. We compared ambulance driving time in two settings: (1) a setting where PCI is only performed at on-site PCI centres, and (2) the current setting that includes on-site and off-site PCI centres.

### Data collection

We obtained a dataset containing the number of inhabitants per four-digit postal code in the Netherlands in 2016 from Statistics Netherlands (CBS). We then used four-digit postal code polygon data describing the geographical coordinates of every Dutch postal code in the same year. We obtained a list of all on-site and off-site PCI centres from the Netherlands Society of Cardiology (NVVC) and the Dutch Association for Thoracic Surgery (NVT). Of note, all PCI centres in the Netherlands perform primary PCI on a 24/7 basis. We collected geographical coordinates of the listed hospitals by entering their addresses into an online coordinate converter (https://www.gps-coordinates.net) (NH). We visually checked the hospitals’ coordinates using a satellite map (https://www.google.com/maps).

### Data and statistical analyses

For the analysis, we used a dataset containing all possible ambulance driving routes between geometric centres (centroids) of four-digit postal codes in the Netherlands in 2015. The calculations were based on a shortest-route algorithm. To estimate the ambulance driving time, we used historical data from the ambulance transportation records and the known speed limit for each road section. We then calculated the ambulance driving time from each four-digit postal code centroid to the centroid of the nearest PCI centre. We assumed that ambulances transporting STEMI patients operate with the highest priority (A1, for possible life-threatening situations). The ambulance driving time was estimated for both on-site and off-site PCI centres. The dataset was produced by an external party (CityGIS, The Hague, the Netherlands) on behalf of the National Institute for Public Health and the Environment (RIVM).

We displayed accessibility to PCI care by plotting the ambulance driving time to the nearest PCI centre from each four-digit postal code centroid on a map. In addition, we combined the ambulance driving time to the nearest PCI centre from each postal code with the number of inhabitants per postal code area. We then plotted the ambulance driving time and the corresponding fraction of the population nearest to an on-site or off-site PCI centre. We compared median driving distances and corresponding interquartile ranges (IQRs) using the Wilcoxon signed rank test. In addition, we used the paired *t*-test to compare means and standard deviations. We assessed the fraction of the population living ≤120 min from a PCI centre. Additionally, we assessed the fraction of the population living ≤60 kilometre (km) from any PCI centre. We used 60 km as a cut-off, because a previous Dutch study demonstrated that a distance of >60 km from a PCI centre is associated with an increased total ischaemic time in STEMI patients [15]. All data and statistical analyses were performed using R (GJK and HG).

## Results

On 1 January 2019, there were 30 PCI centres in the Netherlands: 16 on-site and 14 off-site centres (listed in the Appendix). We calculated 4046 centroids for the Dutch four-digit postal code areas.

### Ambulance driving time

The median ambulance driving time was 18.8 min (IQR 12.2–26.3) when only on-site PCI centres were included and 14.9 min (8.9–20.9) to any PCI centre (*p* < 0.001). In postal code areas with a reduction in ambulance driving time after the expansion of off-site PCI, the median ambulance driving time decreased from 25.4 (18.2–33.1) to 14.7 min (8.9–20.9) (*p* < 0.001). The reduction in estimated driving time after the establishment of a nearby off-site PCI centre ranged from 0.1–67.0 min. Differences in driving time to an on-site or any PCI centre for mainland and non-mainland areas (Wadden Islands) are displayed in Tab. 1.

**Table 1:** Ambulance driving time to percutaneous coronary intervention centres in the Netherlands

	Time to on-site PCI centre (*n* = 16)	Time to any PCI centre (*n* = 30)	*P*-value
*All PC4 areas*			
Median (IQR)	18.8 (12.2–26.3)	14.9 (8.9–20.9)	<0.001
Mean ± SD	21.5 ± 21.0	16.4 ± 18.8	<0.001
*PC4 areas affected by off-site PCI centres*			
Median (IQR)	25.4 (18.2–33.1)	14.7 (8.9–20.9)	<0.001
Mean ± SD	27.8 ± 15.0	15.5 ± 9.2	<0.001
*Mainland PC4 areas*			
Median (IQR)	18.7 (12.1–26.2)	14.8 (8.8–20.8)	<0.001
Mean ± SD	20.3 ± 12.5	15.2 ± 8.0	<0.001
*Non-mainland PC4 areas*			
Median (IQR)	288.6 (111.6–306.2)	288.6 (87.4–306.2)	0.008
Mean ± SD	212.4 ± 98.4	205.0 ± 107.0	0.005

Time in minutes

*PCI *percutaneous coronary intervention,* PC4 *four-digit postal code,* IQR *interquartile range*, SD *standard deviation

### PCI accessibility to the Dutch population

**Fig. 1:**
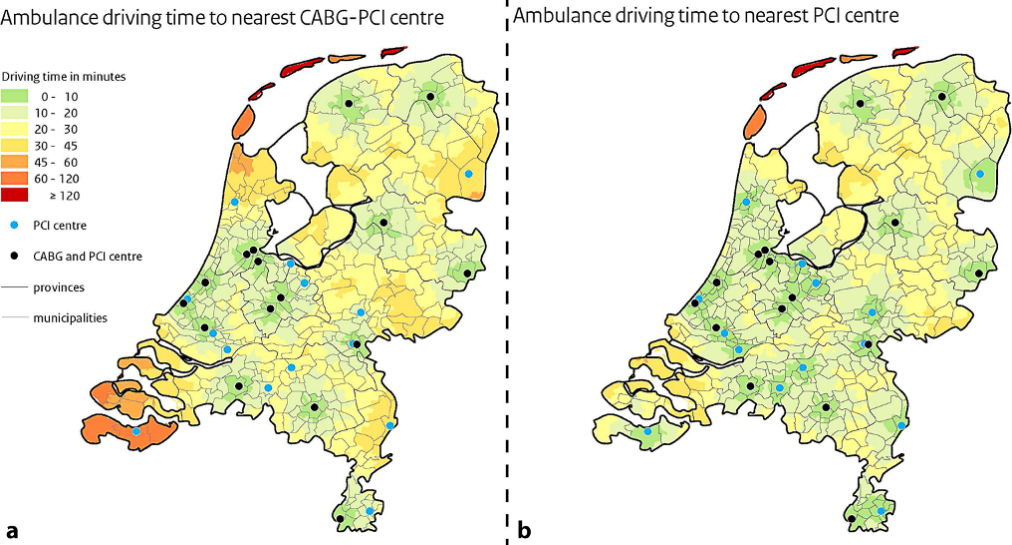
Ambulance driving time to percutaneous coronary intervention (*PCI*) centres in the Netherlands from any Dutch postal code area. **a** Time to nearest PCI centre with on-site surgical back-up for coronary artery bypass grafting (CABG). **b** Current situation in the Netherlands: time to any PCI centre, including off-site PCI centres

Fig. 1 shows the estimated ambulance driving time from each Dutch postal code area to the nearest PCI centre. Patients on the mainland could be transferred to any on-site or off-site PCI centre in ≤120 min. Fig. 1a shows the driving time to the nearest on-site PCI centre, while the ambulance driving time to any PCI centre (on-site or off-site) is displayed in Fig. 1b.

**Fig. 2:**
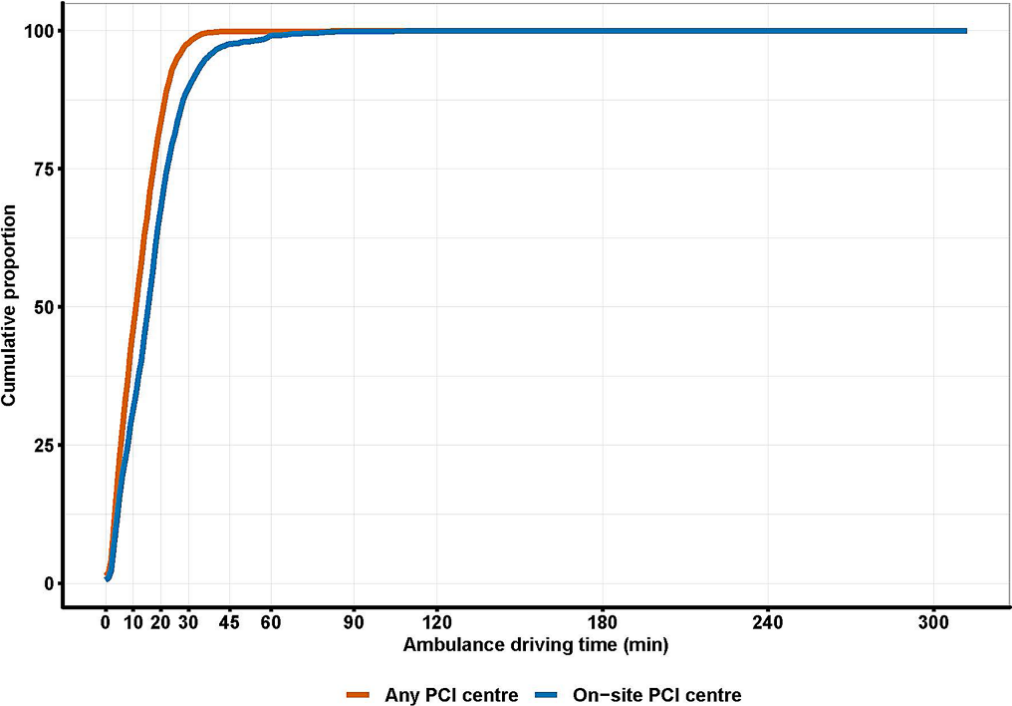
Ambulance driving time to on-site or any percutaneous coronary intervention (*PCI*) centre for cumulative proportion of Dutch population

In 2016, 17,080,825 people were living in the Netherlands, who were served by 30 PCI centres; this amounts to approximately 569,000 individuals per PCI centre. Most of the population (99.81%; *n* = 17,049,945) lived ≤60 km from a PCI centre. The fraction of the Dutch population with access to PCI care in the Netherlands based on ambulance driving time is displayed in Fig. 2. A total of 99.86% (*n* = 17,068,005) of the Dutch population could be transported to an on-site PCI centre <90 min. After the expansion of off-site PCI centres, 99.94% (*n* = 17,080,360) of the population could be transported to any PCI centre <90 min (Tab. 2).

**Table 2:** Time frames for Dutch population to be transported to a percutaneous coronary intervention centre

Ambulance driving time (minutes)	On-site PCI centre (*n* = 16)	Any PCI centre (*n* = 30)
>120	0.040% (6880)	0.040% (6880)
≤120	99.96% (17,085,170)	99.96% (17,085,170)
<90	99.86% (17,068,005)	99.94% (17,080,360)
<60	98.89% (16,902,065)	99.86% (17,068,005)
<45	97.47% (16,659,380)	99.83% (17,063,080)
<30	88.67% (15,155,715)	97.41% (16,648,730)
<20	64.78% (11,072,460)	80.88% (13,823,585)
<10	28.83% (4,926,940)	42.34% (7,236,095)

*PCI* percutaneous coronary intervention

## Discussion

This is the first study to report the impact of off-site PCI centres on accessibility to PCI care on a national scale. Our findings demonstrated that almost every individual in the Netherlands lives within 120 min from a primary PCI centre. In addition, we observed a significant reduction in the median driving distance to any PCI centre, which can be attributed to the expansion of off-site PCI centres.

### Context and interpretation

Our results showed that the current distribution of PCI centres in the Netherlands ensures a high level of accessibility. We demonstrated that almost every individual in the Netherlands lives ≤120 min from a PCI centre (except for inhabitants of certain Wadden Islands). This indicates PCI-mediated reperfusion rather than a pharmacoinvasive strategy can be provided to all Dutch STEMI patients, in accordance with European Society of Cardiology guidelines [4]. Moreover, 99.9% of Dutch inhabitants could be transported to a PCI centre in <60 min and 97.4% in <30 min, which can contribute to adherence to the European recommendations of a target STEMI diagnosis-to-wire crossing time of ≤90 min.

A large Swedish study of >10,000 STEMI patients demonstrated that a first medical contact-to-PCI delay of >1 h is associated with an increase in mortality (first medical contact-to-PCI 61–90 versus <30 min: hazard ratio 1.57; 95% confidence interval 1.03–2.41) [16]. Therefore, the short transportation delays in the current study indicated that the current distribution of PCI centres contributes to the quality of care and outcome of ACS patients in the Netherlands.

We observed that 99% of the population lived ≤60 km from a PCI centre. This cut-off was based on a study of >4000 STEMI patients from a large on-site PCI centre in the Netherlands [15]. This study demonstrated an independent association between ischaemic time and driving distance >60 km among STEMI patients who first present at a non-PCI ‘spoke’ centre. However, this finding was not observed in patients for whom the driving distance was >60 km and who immediately called the ambulance services. Possibly, a transportation delay does not increase ischaemic time among ambulance patients in a small and densely populated country such as the Netherlands, because this delay is balanced by the additional preparation time of the catheterisation laboratory at the PCI centre, which contributes to a shorter door-to-balloon time. In Sweden, approximately 55% of the population live <60 km from an on-site PCI centre and approximately 75% of the population are <60 km from any 24/7 PCI centre [17]. However, due to several country-specific differences, including geographical differences, caution should be used when directly comparing the two countries and their healthcare systems. Moreover, despite longer driving distances to PCI centres, STEMI care in Sweden is regarded among the best in the world [18, 19].

The number of off-site PCI centres has increased, and these centres are regarded as safe when compared with on-site PCI centres [11, 12, 20, 21]. In the UK, emergency cardiac surgery after off-site PCI occurs in <0.1% of patients [12]. Time delay in STEMI patients significantly decreased at two individual Dutch hospitals after they started offering off-site PCI [13, 22]. These findings support off-site (primary) PCI. Conversely, Denmark has implemented a more centralised approach. Only four primary PCI centres serve approximately 5.5 million individuals (±1.4 million inhabitants per centre), after two high-volume PCI centres merged into one very large centre performing 1000 primary PCIs annually [23]. After this merger, quality of care was maintained, and the symptom-to-balloon time was decreased by 30 min (*p* < 0.001). Moreover, a Danish nationwide study could not demonstrate an association between distance to a PCI centre and improved outcome in out-of-hospital cardiac arrest patients, supporting the centralised strategy [24].

### Implications

Ten years ago, the expansion of off-site primary PCI centres in the Netherlands sparked reactions, from both supporters and opponents [25, 26]. Our results showed off-site PCI centres improve accessibility to PCI care. It seems unlikely that additional expansion of off-site PCI will further benefit the accessibility. This year, the Dutch Healthcare Inspectorate (IGJ) reported that every off-site PCI centre is able to perform the minimum number of 600 PCIs per year [27]. However, some off-site PCI centres are unable to guarantee 24/7 primary PCI care. Moreover, off-site PCI centres were ordered to improve their collection of procedural and outcome data, which will be overseen by the recently established Netherlands Heart Registry.

### Strengths and limitations

One of the strengths of this study is the use of reliable data from government institutions to display a simple structure indicator reflecting one aspect of the quality of a healthcare system [28].

However, there are some limitations. First, our study focused on the impact of off-site PCI expansion on time delays in STEMI patients. However, other local initiatives that were concomitantly introduced, such as prehospital ECG transmission or a home-to-hospital feedback dashboard, have also led to shorter time delays [29, 30]. Second, we used historical ambulance transportation records to build a model to estimate ambulance driving time. Therefore, this study does not fully reflect real-life clinical practice since we did not account for local practice agreements, for example rotating on-call PCI centres in the Rotterdam region. Third, we estimated ambulance driving times for non-mainland areas. However, since 2016, STEMI patients in these areas can be transported by a helicopter, which significantly reduces delays [31]. Fourth, we did not assess the impact of off-site PCI centres on individual patient outcomes. Fifth, we have only presented results from the Netherlands, a small country with a tight network of general hospitals and PCI centres and without any significant geographical barriers than can complicate logistics. Finally, we did not account for individuals residing close to the borders who may undergo PCI in Belgium or Germany.

## Conclusion

Based on a computational model, the expansion of off-site PCI centres in the Netherlands has reduced the ambulance driving time to PCI centres in this country. It seems unlikely that additional expansion will further improve accessibility to PCI. Future efforts to improve PCI and ACS care should focus on strengthening the collaboration between general practitioners, ambulance services, and off-site and on-site PCI centres, and on further development of the Netherlands Heart Registry.

## Appendix

### List of on-site PCI centres

Amphia Ziekenhuis, location Molengracht (Breda); Amsterdam University Medical Center, location Amsterdam Medical Center and location VU University Medical Center (Amsterdam); Erasmus Medical Center (Rotterdam); Catharina Ziekenhuis (Eindhoven); HagaZiekenhuis, location Leyweg (Den Haag); Isala (Zwolle); Leiden University Medical Center (Leiden); Maastricht University Medical Center+ (Maastricht); Medical Centre Leeuwarden (Leeuwarden); Medisch Spectrum Twente (Enschede); Onze Lieve Vrouwe Gasthuis, location Oost (Amsterdam); Radboud University Medical Centre (Nijmegen); St. Antonius Ziekenhuis (Nieuwegein); University Medical Center Groningen (Groningen); University Medical Center Utrecht (Utrecht).

### List of off-site PCI centres

Albert Schweitzer Ziekenhuis, location Dordwijk (Dordrecht); Canisius-Wilhelmina Ziekenhuis (Nijmegen); Elisabeth-TweeSteden (Tilburg); HMC Westeinde (Den Haag); Jeroen Bosch Ziekenhuis (Den Bosch); Maasstad Ziekenhuis (Rotterdam); Meander Medical Centre (Amersfoort); Noordwest Ziekenhuisgroep, location Alkmaar (Alkmaar); Rijnstate Ziekenhuis (Arnhem); Tergooi, location Blaricum (Blaricum); Treant Ziekenhuis, location Scheper (Emmen); VieCuri Medical Centre (Venlo); ZorgSaam Zeeuws-Vlaanderen (Terneuzen); Zuyderland Medical Centre, location Heerlen (Heerlen).
